# Mental health and disability research priorities and capacity needs in Ghana: findings from a rapid review and research priority ranking survey

**DOI:** 10.1080/16549716.2022.2112404

**Published:** 2022-09-29

**Authors:** Benedict Weobong, Kenneth Ae-Ngibise, Grace Mwangi, Lionel Sakyi, Crick Lund

**Affiliations:** a Department of Social and Behavioural Sciences, College of Health Sciences, University of Ghana, Accra, Ghana; b Operations Research and Global Learning, Ghana Somubi Dwumadie (Ghana Participation Programme), Accra, Ghana; c Kintampo Health Research Centre, Research and Development Division, Ghana Health Service, Accra, Ghana; d Tropical Health, London, UK; e Centre for Global Mental Health, Health Service and Population Research Department, Institute of Psychiatry, Psychology and Neuroscience, King’s Global Health Institute, King’s College London, London, UK; f Alan J Flisher Centre for Public Mental Health, Department of Psychiatry and Mental Health, University of Cape Town, Rondebosch, South Africa

**Keywords:** Research priorities, mental health, disability, low and middle-income country, Ghana

## Abstract

**Background:**

Identification of national research agendas for mental health and disability can be supported by well-designed research priority-setting studies. Few low- and middle-income countries (LMICs) have undertaken such studies.

**Objective:**

To identify mental health and disability research priorities in Ghana.

**Methods:**

A mixed methods study comprising a rapid review, research priority ranking survey, and research capacity needs assessment survey was employed. Participants in the surveys included five expert pools identified from online search and existing database on mental health civil society organisations/non-governmental organisations. The research priority ranking was completed in two stages, using the Child and Nutrition Research Initiative (CHNRI) method to identify priority questions for immediate and short term (0 to 5 years) and medium to long term (>5 years) in stage two. Both surveys were deployed online using google forms. Analysis for the ranking survey involved computing total scores from the CHNRI criteria and generating ranks for the research questions.

**Results:**

A total of 68 experts (97% response rate), generated 94 and 92 questions for the short and long term, respectively. Forty experts (58% response rate) completed the ranking stage. The top 10 ranked research questions included: 4 questions addressing health systems; 2 questions on epidemiology; and 4 questions on interventions. All research questions were considered urgent and should be conducted in the immediate to short term (0–5 years). The methodological capacity of researchers to conduct disability and mental health research is weak.

**Conclusion:**

Our approach has generated an agenda for mental health and disability research priorities for Ghana and demonstrated that it is feasible to employ a systematic methodology for research priority setting that includes key parameters of context and research capacity.

## Background

Mental, Neurological and Substance Use (MNS) [1] conditions are key drivers of increased morbidity and mortality in the world over [2]. The burden of these conditions has been increasing in main low-and-middle-income countries (LMIC) [3]. While these increases are being recorded, most people living with MNS conditions do not receive the required treatments, and in Ghana, this treatment gap is estimated to be as high as 98% [4]. This neglect is primarily due to the low priority that has been accorded mental health in the public health agenda [5].

Research on mental health is increasingly recognised as integral to the strengthening of national health systems [6] to respond to the treatment gap. The huge disparity in research investment was identified several decades ago and is referred to as the 10/90 gap: less than 10% of global funding for research is spent on diseases that affect more than 90% of the world’s population [7,8]. Despite some successes, this trend remains, particularly for mental health [3]. These disparities in research will pose significant challenges in achieving the sustainable development goals [9]. There is an urgent need to develop national research agendas that align with knowledge gaps [6].

The identification of national research agendas for mental health and disability can be supported by well-designed research priority-setting studies. Priority setting exercises are promising for surfacing vital evidence [10]. They are an important step in identifying the most pressing mental health challenges in a given setting [11,12], in order to address the 10/90 gap and ensure the most efficient use of resources. These studies have largely been carried out at global or regional levels [13], and at the level of the broad area of health, but as argued in other reports, priority-setting methodology needs to reflect the context [14], country-specific needs [15], and be iterative [16]. The field of health research priority setting is relatively new and few LMICs have established these processes [17,18], particularly for mental health. There are however notable initiatives such as the WHO’s Global Health R&D Observatory [19], Lancet Commission on Global Mental Health [3], and Grand Challenges in Global Mental Health [20]. In the field of mental health, there have been very few reported country-level research priority setting initiatives [21,22]. In Ghana, there have been previous attempts by the Mental Health Authority of Ghana [23] and the Ghana Health Service [24] to identify priorities for health research, but these were primarily for operational purposes and thus were not peer-reviewed and published. Important limitations were noted, including the lack of clarity in the selection of stakeholders. The proper selection of stakeholders is important because it is difficult to judge the validity of the priorities identified without adequate stakeholder involvement [14]. The Government of Ghana passed a Disability Act in 2006 (Act 715 [25]) and ratified the Convention on the Rights of Persons with Disabilities in 2012, affirming its recognition of the rights of persons with disabilities. The Act stipulates the establishment of a National Council on Persons with Disability (NCPD). A key function of NCPD is to promote studies and research on issues of disability and provide education and information to the public on issues of disability, but we are not aware of a clear research agenda to guide the conduct of research.

Ghana Somubi Dwumadie (Ghana Participation Programme) is a four-year disability programme in Ghana, with a specific focus on mental health. This programme is funded with UK aid from the UK government [26]. The conduct of research priority setting studies for mental health and disability is one of the key strategies for influencing change.

There are several published methods describing different approaches for setting priorities for health research, but a common observation is that there is no single best practice [27,28]. The Child and Nutrition Research Initiative (CHNRI) method [29], is arguably the most used approach in setting mental health research priorities [21]. The Combined Approach Matrix (CAM) [30] has also been used, but unlike CHNRI, it does not follow a standard prioritization process [31]. The Council on Health Research and Development (COHRED) method [28] combines elements of CHNRI and CAM. However, little is known about how to strengthen the standard research prioritization process with key contextual information such as the level of research activity, capacity, and funding. It is also not clear if the individual CHNRI criteria will receive equal endorsement in terms of usefulness in determining research priorities in the Ghanaian context. The lessons from this enhanced process are transferable and can inform other African countries who may wish to conduct similar priority setting studies, particularly for mental health.

The aim of this paper was to identify mental health and disability research priorities for Ghana within the context of the prevailing research ecosystem.

## Methods

**Figure 1.**
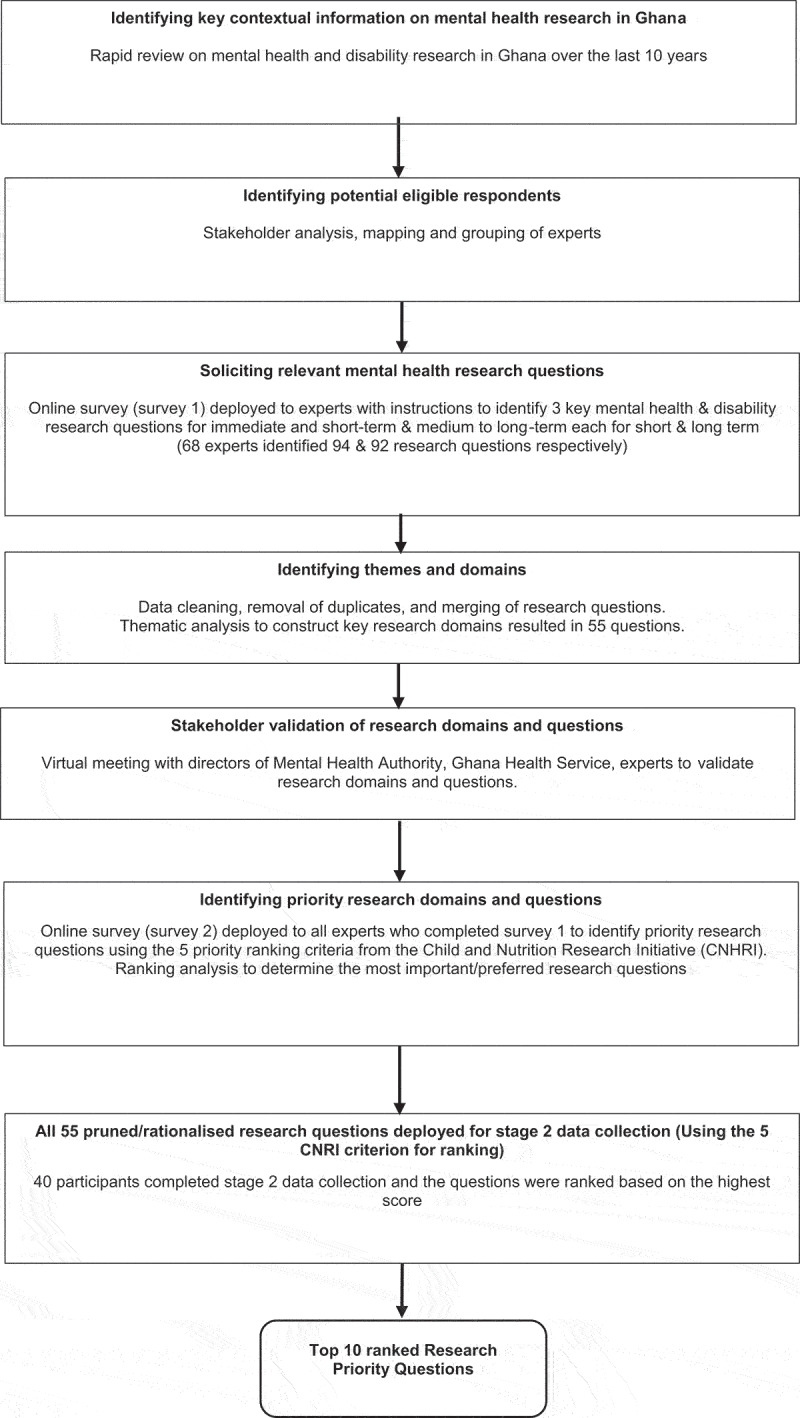
Flowchart of mental health and disability research priority ranking process.

Study design. Two study designs were employed: first, a rapid review on mental health and disability research in Ghana over the last 10 years; and second a structured cross-sectional survey on priority mental health and disability research questions for the short to medium term and long term. Ethical approval for the study was obtained from the Ghana Health Service Ethics Review Committee (GHS-ERC025/08/20) and King’s College London Research Ethics Committee (LRS-20/21-20,866). The two studies are described below. The schedule of activities and steps is shown in Figure 1.

### Study 1: rapid review

#### Search strategy

Standard rapid review strategies [32] were employed. The detailed methodology on the rapid review is provided in our companion paper (in preparation). In summary, a comprehensive search of electronic databases (PubMed, Embase, PsycINFO, Scopus and Cochrane) was done to identify relevant studies on mental health and disability conducted in or on Ghana over the 10 years from 2010 to 2020. The key search terms used in this review are ((‘mental health’ [MeSH Terms] OR (‘mental health’ [MeSH Terms] OR (‘mental’ [All Fields] AND ‘health’ [All Fields]) OR ‘mental health’ [All Fields])) OR ‘disability’ [All Fields]) AND (‘Ghana’ [MeSH Terms] OR ‘Ghana’ [All Fields]). The full search strategy is included in the appendix. Reference lists of selected articles and relevant systematic reviews were screened to identify any other relevant articles that were missed during the initial database search. All types of studies were included regardless of the study design and the study population. Following a rigorous selection, 375 articles were retrieved, and the relevant data extracted using a standard data extraction form.

### Study 2: MH research priority ranking survey

Design: This was an iterative cross-sectional survey conducted over two stages.

Stage 1: Identification of relevant research questions

This stage involved two key steps as described below.

Step 1: Stakeholder mapping and grouping process: Study participants were identified through a process of stakeholder mapping and grouping to ensure a broad range of respondents for the study. The mapping exercise was conducted through online searches and a personal database maintained by a colleague who has been conducting various ethnographic mental health research in Ghana for the past 15 years [33] on mental health service providers to identify potential experts in the following pools: (a) Expert pool 1: Clinicians; (b) Expert pool 2: Researchers/academia; (c) Expert pool 3: Non-Governmental Organisations/Civil Society Organisations in mental health and disability; (d) Expert pool 4: Policy makers; and (e) Expert pool 5: Funders, multilateral and unilateral organisations. Our stakeholder mapping identified and oversampled 153 potential respondents. However, based on the response rate from similar previous surveys [21], we anticipated up to 70 complete responses.

Step 2: Data collection and processing: An online survey was conducted from 6 November to 20 November 2020. Respondents from all the expert pools were asked to provide responses to two questions on research priorities: (1) please identify your top 3 research questions in mental health and disability in the **immediate and short term (0–5 years**); and (2) please identify your top 3 research questions in mental health and disability in the **medium to long term (>5 years).**

In order to explore current research capacity needs in relation to the identified research priorities in Ghana, we derived a set of research domains developed for a mental health research capacity needs assessment in six low- and middle-income countries as part of the Emerging Mental Health systems in low- and middle-income countries (Emerald) project [34]. These questions were deployed only to respondents from expert pool 2 (researchers/academia) and the leadership of research/academic institutions. Participants were asked to respond to two separate but interlinked questions. In the first question, participants were asked to rate (on a 5-point Likert scale; 1 = very weak; 2 = weak; 3 = OK but could be improved; 4 = Strong; 5 = very strong) the current institutional capacity to conduct research in 16 pre-selected areas: Health Services Research; Disability Research; Global Mental Health Research; Qualitative Research; Population Level Surveys; Economic Evaluation; Evaluation of Complex Interventions; Analysis of complex population datasets; Health economics evaluation; Implementation science; Empowering service users in research; Action Research; Research on stigma and discrimination; Public engagement; Ethical conduct of research; and Social and Behaviour Change Communication. In the second question, participants were asked to rate (on a 3-point likert scale; 1 = low priority; 2 = moderate priority; 3 = high priority), the level of priority for building capacity to conduct research in the above pre-selected areas. We had planned to conduct qualitative interviews with relevant stakeholders to elicit views on the interface between policy and research, key challenges for research capacity, and priorities for development but this was not done because of the challenges posed by COVID-19.

Data were downloaded onto an Excel spreadsheet and transferred to STATA for analysis.

In stage 1 of the survey, 68 participants (97.0% response rate) identified 186 research questions comprising 94 and 92 for the immediate, short-term, and medium-to-long-term Mental Health and Disability Research Priorities, respectively (Supplementary Table 1). An examination of the two sets of questions by timeline showed that the same set of questions were noted for both timeframes. These were thus combined, and a uniform set of questions taken forward to the ranking stage. The investigators (CL, AK, LS, BW) pruned and rationalised the research questions, removing duplicates, merging the same or similar questions, and restructuring statements into questions. This process resulted in 55 mental health and disability research questions, which were grouped in five thematic areas (Supplementary Table 2) and taken forward to stage 2 for scoring and ranking.

### Stage 2: scoring and ranking of research questions

In the second stage, the rationalised 55 research questions were emailed to all 68 stakeholders that participated in the first survey via Google Forms to score based on the Child and Nutrition Research Initiative (CHNRI) criteria. The CHNRI is a priority-setting methodology developed by Rudan et al. (2007) that had been previously used to establish mental health priorities on a global level [35]. The CHNRI criteria include: (1) the likelihood of answerability in an ethical way, (2) the likelihood of efficacy and effectiveness, (3) the likelihood of deliverability and affordability, (4) the maximum potential for disease burden reduction, and (5) the likely impact of equity in the population. Thus, for each research question, participants were asked to rate the relevance of each criterion by selecting one of the following response options: 1. ‘Yes’, 2. ‘No’, 3. ‘I Don’t know’ or 4. ‘Not Applicable’. Given these criteria were applied for the first time in the Ghanaian context, the study examined the applicability of the criteria in terms of their individual usefulness (measured by frequency of endorsement) in identifying research priorities. Additionally, participants were asked to indicate if a particular research question should be addressed within the immediate and short term [0–5 years] or medium to long term [>5 years].

More than half (40/68) of participants returned completed questionnaires. The rationalised 55-question list is presented in supplementary Table 2. All the included questions received at least five ratings based on the 5 CHNRI criteria described.

### Analysis

#### Estimation of score for ranking

For this analysis a ‘Yes’ response to any of the 5 CHNRI criteria was coded 1, and all other response options coded 0. Thus, for each research question, the total possible score is 5. This translates to a total score of 275 for the 55 questions. Scores across each respondent were summed and a total score and proportion generated for each research question. Mean scores for each question were also computed and reported along with the score proportion. The mean scores and proportions were ranked, and the top 10 research questions identified.

#### Estimation of most endorsed CHNRI criteria

Similarly, for this analysis, a ‘Yes’ response to any of the 5 CHNRI criteria was coded 1, and all other response options coded 0. Thus, across respondents the total possible score for each CHNRI criteria is 40. Scores for each CHNRI criteria were summed across respondents and a total score generated for each research question. Mean scores were generated for each CHNRI criteria across all questions, and test of difference in means conducted.

#### Determination of mean existing research capacity and priority score

In order to determine the current research capacity needs in relation to the identified research priorities in Ghana, mean scores and standard deviations were generated for existing research capacity over a range of scores from 1 to 5 for each of the 16 pre-identified research areas. A similar analysis was conducted to generate mean scores and standard deviations to identify priority research domains across each research area.

Additional chi-square analysis was conducted to determine differences in demographic characteristics between participants in stage 1 (survey 1) and stage 2 (survey 2). Probability values of p < 0.05 were considered to be statistically significant differences that could not have been due to chance.

## Results

### Rapid review

Key relevant findings from our rapid review are provided. The detailed results on the rapid review are provided in our companion paper (in preparation). In summary, of the 375 articles included in this review, 232 (62.0%) of the articles were on mental health, while the remaining 143 (38.0%) were on disability.

#### Mental health research output and funding

**Figure 2.**
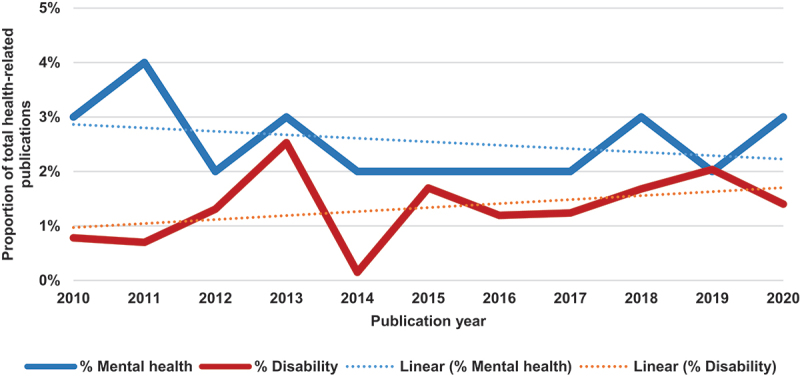
Mental health and disability research publications as a proportion of total health-related publications over a 10-year period.

Figure 2 shows that within the ten-year period (2010–2020), there has been a general decline in reported studies on mental health as a proportion of total health-related research publications. The current trend however shows an increasing trajectory between 2019 and 2020. Supplementary figure 1, however, shows that within the same period, there has been a steady increase in absolute numbers of reported studies on mental health in Ghana. Comparatively, there were more studies on mental health than studies on other disabilities.

The review also identified 13 key themes covered in mental health research. Of these, a significant majority (69.0%) focused on the epidemiology (prevalence, determinants) of mental health conditions and events. On the contrary, very little research has been conducted around health policy and systems (5.0%), including mental health workforce, service delivery collaboration (with traditional and faith-based healers), and policy and legislation.

In terms of design, most of the mental health studies included in this review were either observational quantitative studies (n = 130, 56.0%) or observational qualitative studies (n = 79, 34.0%). There were very few interventional studies (n = 6, 3%). In terms of research funding, 98 (42.0%) of the articles were from studies that had received external funding support, and 16.0% were unfunded.

#### Other disabilities research output and funding

Figure 2 shows that within the ten-year period (2010–2020), there has been a steady increase in reported studies (N = 143) on disability (excluding mental health) as a proportion of total health-related research publications. This observation is consistent with trends in the actual number of studies conducted on disabilities within the same period (supplementary figure 1), with a clear increase recorded between 2016 and 2019.

Similar to the profile of studies on mental health, understanding the epidemiology (prevalence, determinants) of disability in Ghana was the most common (54.0%) research theme identified in the scoping review. Few studies have researched on issues of stigma and discrimination (7.6%), and no reported research was conducted on the implementation of policies and rights of persons with disabilities as stipulated in the 2006 Ghana’s Disability Act 715.

Further, most of the studies were observational in design (n = 70, 49.0%), and there were no experimental studies. Forty-eight (34.0%) studies had persons with disabilities as the primary study subjects. A significant number of studies had also focused on caregivers as the primary subjects (n = 25, 17.0%) and their role in providing care for persons with disabilities. In terms of sex, 130 (91.0%) articles were all gender inclusive (i.e. study participants included women and men), 13 (9.0%) articles had women only study participants, and no study was focused on men only. Fifty-two (36.4%) disability articles were from studies that were funded from external sources, and 24 (17.0%) were unfunded.

### Research priority ranking surveys

#### Characteristics of participants

Table 1 presents the demographic characteristics comparing participants who completed the first stage and those who completed the second stage. Participants across the two surveys had similar characteristics, except the type of expert; stage two had significantly fewer experts in the clinician pool. Across both surveys, there were significantly more participants aged between 25 and 44 years, with more males than females. The majority had a post-graduate education up to diploma or masters. A significant majority of the experts were clinicians, with 1 in 5 from Non-Governmental Organisations or civil society organisations.

**Table 1.** Socio-demographic characteristics of study participants in first and second-stage surveys.

Variable	Category	Participants		P-value
		**Stage 1 survey** **[N = 68 (%)]**	**Stage 2 survey [N = 44 (%)]**	
Age group	25–34 35-44 45–54 55-64	27 (39.7) 28 (41.2) 9 (13.2) 4 (5.9)	12 (30.0) 16 (40.0) 8 (20.0) 4 (10.0)	0.571
**Sex**	Male Female	42 (61.8) 26 (38.2)	31 (77.5) 9 (22.5)	0.824
**Highest Education Achieved**	Membership in Psychiatry Bachelor’s degree Diploma MD Masters PhD	1 (1.5) 12 (17.6) 29 (42.6) 1 (1.5) 21 (30.9) 4 (5.9)	1 (2.5) 7(17.5) 11 (27.5) 0 (0) 17 (42.5) 4 (10.0)	0.293
Expert pools	Academic/Research Clinician/Psychiatrist/Psychologist Donors/Multilaterals/Unilaterals NGOs/CSOs Policy	5 (7.4) 42 (61.7) 5 (7.4) 13 (19.1) 3 (4.4)	5 (12.5) 19 (47.5) 4 (10.0) 10 (25.0) 2 (5.0)	0.004

#### Priority research areas

Table 2 summarises the top 10 priority research questions, the corresponding broad research themes, CHNRI criteria scores, and recommended timelines for addressing the research questions. The majority of the top 10 priority research questions focused on health systems and interventions research with specific questions on scale-up of mental health and disability services. Experts also prioritised epidemiological questions to understand the consequences of stigma, and social determinants of mental health. Research questions around public health emergencies, such as COVID-19 and community engagement did not feature in the top 10 priority questions.

**Table 2.** Top 10 priority research questions and their thematic areas for Ghana.

		Individual CNRI criteria score (0–40)					Time frame	
Thematic area *(Research Question/s)*	% total score (275)/mean score	Ethical	Efficacy	aDeliv	Burden	Equity	Immediate to short-term n (%)	Medium to long-term n (%)
**Health systems, policy and mental health and disability legislation studies** in order to unpack human-rights issues and their impact on mental health and disability; and integrate mental health in primary health care *(How can we scale up mental health and disability services in Ghana?)*	171 (62.2%)/4.28	35	35	31	34	36	24(60)	16(40)
**Design and evaluation of intervention studies, including economic evaluation***(What are the ways of improving working conditions for mental health professionals in Ghana?;* *What livelihoods and psychosocial support activities are most appropriate and effective for people with disabilities?;* *What interventions and educational programmes will aid in improving mental health literacy to reduce stigma and protecting the rights of people living with mental illness and disabilities?)*	168 (61.1%)/4.20	36	35	34	32	31	28(70)	12(30)
	168 (61.1%)/4.20	35	33	34	33	33	29(72.5)	11(27.5)
	166 (60.4%)/4.15	34	31	34	33	34	20(51.3)	19(48.7)
**Epidemiological studies** on incidence and prevalence of mental health conditions and to identify how mental health conditions emerge, what makes people more susceptible or resilient than others, consequences *(How is mental health stigma impacting on the economic livelihoods of persons with psychosocial disabilities?)*	164 (59.6%)/4.10	35	33	29	34	34	25(62.5)	15(37.5)
**Health systems, policy and mental health and disability legislation studies** in order to unpack human-rights issues and their impact on mental health and disability; and integrate mental health in primary health care *(What proportion of people with mental health and disability related health care needs access the services they require, and do not suffer financial hardship as a result? How does this differ by different groups: men/ women, rural/ urban, rich/ poor, etc?* What are the causes of stigma and discrimination against people with mental illness and what steps can be taken to address it?)	162 (58.9%)/4.05	33	32	29	34	34	26(65)	14(35)
	161 (58.5%)/4.03	35	30	28	34	34	26(66.7)	13(33.3)
**Epidemiological studies** on incidence and prevalence of mental health conditions and to identify how mental health conditions emerge, what makes people more susceptible or resilient than others, consequences *(How does extreme poverty and other social determinants (including domestic abuse, childhood adversity) exacerbate mental health conditions in Ghana?)*	161 (58.5%)/4.03	34	32	28	33	34	24(60)	16(40)
**Design and evaluation of intervention studies, including economic evaluation (***What are the benefits and challenges of delivering mental healthcare through community-based instead of institutional care and what impact does the removal of institutional care have on mental healthcare in Ghana?)*	160 (58.2%)/3.97	33	33	30	33	31	24(60)	16(40)
**Health systems, policy and mental health and disability legislation studies** in order to unpack human-rights issues and their impact on mental health and disability; and integrate mental health in primary health care *(What is the importance of community mental health officers in mental health delivery?)*	159 (57.8%)/3.87	33	32	31	32	31	24(60)	16(40)
**Mean CNRI criteria score (n = 55)**	-	30.4	31.7	32.2	32.3	33.5		

^a^Deliverability

Participants assigned almost equal importance to the five CHNRI priority rating criteria as applied to each research question. All of the top 10 research questions were considered urgent and should be conducted in the immediate and short term (0–5 years).

### Priority mental health conditions

Participants in survey 2 also identified mental health conditions that need to be prioritised for research in Ghana. Almost half (47.5%) of the participants identified all the major mental, neurological and substance [1] use conditions as needing equal prioritisation for research. However, for specific MNS conditions, research on depression (17.5%) was most prioritised, followed by substance use disorder (12.5%), epilepsy (10%), bipolar disorder (7.5%), and Schizophrenia (5%).

### Research capacity survey

**Figure 3.**
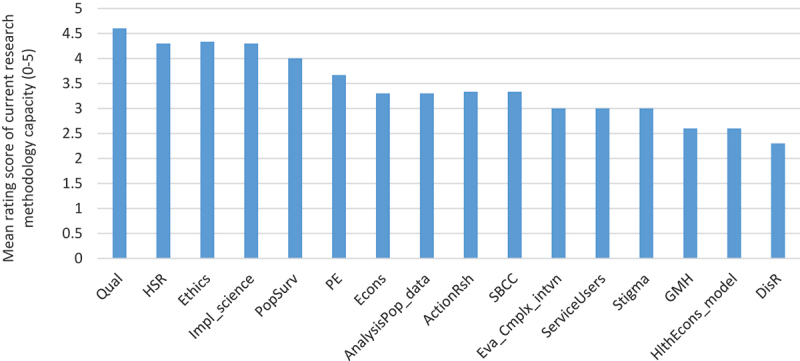
Distribution of mean research capacity score by research domains.

Directors of two of the three health researcher centres of Ghana Health Service, and a senior faculty member of a public university in Ghana provided responses for this survey. Figure 3 shows the mean research capacity score for each research domain. Six out of the 16 domains assessed were rated as weak to average (</ = 3) capacity for research. The remaining 10 domains were rated as above average to strong. As illustrated in the progressively decreasing bars, capacity for disability research (mean = 2.3; SD = 1.15), global mental health research (mean = 2.6; SD = 0.57), and health economics modelling (mean = 2.6; SDD = 1.15) was particularly weak.

Key:HSR: *Health Services Research*; DisR: *Disability Research*; GMH: *Global Mental Health Research*; Qual: *Qualitative Research*; PopSurv: *Population Level Surveys*; Econs: *Economic Evaluation*; Eva_Cmplx_intvn: *Evaluation of Complex Interventions*; AnalysisPop_data: *Analysis of complex population datasets*; HlthEcons_model: *Health economics evaluation*; Impl_science*: Implementation science*; ServiceUsers: *Empowering service users in research*; ActionRsh: *Action Research*; Stigma: *Research on stigma and discrimination*; PE: *Public engagement*; Ethics: *Ethical conduct of research*; SBCC: *Social and Behaviour Change Communication*Red bars: illustrating mean score values <3 (weak research capacity)

## Discussion

The top ten research priorities ranked by answerability, effectiveness, deliverability, equity and potential impact on burden of mental health conditions for Ghana include understanding the social determinants of mental health, scale-up and equitable access including human resources for mental health and disability services, and interventions for mental health and disability. These were identified within a context of constrained mental health and disability research output, informed by poor research methodology capacity, and slow increase in funding.

The gamut of illustrative research questions or aims can be summarised under three broad themes. First, the results underscore the importance of strengthening the understanding of the epidemiology of mental health conditions in Ghana. Questions around the aetiology of mental health conditions, with a particular emphasis on the social determinants of these conditions are recommended as needing immediate attention. Understanding and properly researching the epidemiology of mental health and other disabilities is an important goal because of the established links between physical and mental health; 46% of the people with a mental health condition also have a long-term physical condition, and the reverse is also well documented with 30% with long term physical conditions having a mental health condition [36]. But also, because we still do not know how co-morbidities interact with each other and with social factors [10]. These multi-morbidities have economic costs [37,38]. Second, our results strongly support a health systems and policy approach to mental health services. Experts supported urgent research that supports the scale-up of mental health and disability services. An important related question worth researching is how to improve equitable (sex/place of residence/socioeconomic status) access to mental health and disability services. This is also supported by the findings from our rapid review that suggest the lack of studies that examine disaggregated effects, and few studies (only four studies) that address challenges with the woefully inadequate mental health workforce in Ghana. These are important goals for Ghana and many LMICs given the widely recognised major challenges in reaching all the people who need care and support, as well as enabling them timely access to evidence-based treatment and support [10,39]. Research in scaling up mental health and disability services would lessen the effect of stigma and discrimination on timely access to mental health services [40].

Experts further recommended research questions around human resources for mental health services delivery. For example, the role of cadres of mental health professionals, such as community mental health officers in providing mental health services was identified as an important research question. Third, our results clearly identify the key role of intervention development, implementation, and evaluation as a priority research area in Ghana. Experts were particularly keen on research that compares the effectiveness of community-based versus facility-based approaches for the treatment and management of mental health conditions. These priority areas of research for Ghana are in step with the current thinking of Global Mental Health; the thematic areas align with recommendations from the Grand Challenges of Global Mental Health research report [20], and the Lancet Commission on Global Mental Health and Sustainable Development [3]. The priority research thematic areas identified for Ghana are consistent with other reports from South America (Brazil and Chile) [21,22], and importantly, also the mental health research agenda policy of the Mental Health Authority of Ghana. To illustrate, in the Brazil study, 4 out of the top 10 ranked research priorities relate to intervention studies (effectiveness, cost-effectiveness, and policy); intervention studies were also identified as high priority in Ghana. A notable point of difference is the prioritisation of research on mental health and disability policy and legislation with a focus on human-rights; this was an explicit priority for Ghana but not Brazil and Chile. The interest in research around human rights in Ghana was probably influenced by WHO’s quality rights in mental health project that has attracted a good amount of interest in Ghana [41].

The results of this priority-setting agenda for Ghana are to a large extent reflective of the mental health and disability research profile of Ghana for the 10-year period between 2010 and 2020. Our rapid review and research capacity survey show clear gaps that can benefit from the formulated research agenda in this study. First, peer-reviewed mental health and disabilities research has not improved since 2010, and the research agenda provides an important framework to help reverse this trend in the next 10 years. Second, the priority for intervention studies if heeded will be important to address the deficit of intervention studies, currently at only 3% for mental health and 0% for disabilities research. Third, and quite relevant to point two, there is a clear lack of both methodological and local funding capacity to design and conduct Global Mental Health and disability research in Ghana; the research agenda identifies some priorities if this trend is to be reversed in the next 10 years.

Our study has several strengths. This is the first study in Ghana to systematically document and report on a priority-setting agenda, adopting a rigorous participatory methodology. Priority-setting methodology needs to reflect the context [14] and country-specific needs [15,16], and our rapid review provided this crucial information and a useful validation of the identified priority areas; previous priority-setting studies have been unable to do this. We also addressed the issue of time-framing as an important parameter in judging the urgency with which to tackle important research questions; this is additionally useful for planning around allocation of funding for research. Further, we validated the findings from the first step of the priority-setting survey and the rapid review with key stakeholders in a workshop before proceeding to the ranking stage. In addition, our selection of experts was systematic and based on an existing local directory of mental health civil society organisations and NGOs. Finally, we employed the CHNRI methodology, an objective and widely used priority ranking tool. The CHNRI criteria have content validity for use in the Ghanaian context and are assigned equal importance in determining mental health and disability research priorities.

Nevertheless, we note several limitations for course-correction in the future. First, the response rate (58%) for the priority ranking was encouraging compared to other similar studies in Brazil (56%) and Chile (36%) but not optimal. The potential for response bias and a threat to the internal validity of our results cannot be ruled out. Second, in our attempt to reduce the number of initial submissions of research questions and improve the response rate for the ranking survey, the research team pruned and grouped questions. We may have missed important questions and thus introduced a systematic error in the choice of response options. Third, we were unable to follow through with our planned qualitative interviews with experts for the research capacity survey. This was entirely due to COVID-19 restrictions at the time. These limitations notwithstanding, our approach is a significant improvement over previous studies, and the first report of this kind in the sub-Saharan Africa region.

## Conclusion

We conclude with thoughts on the approach and implications. Our approach to the research priority-setting agenda for Ghana shows it is feasible to employ a systematic participatory methodology that includes key parameters of context and research methodology capacity. We encourage maintaining this process of setting research priorities. The implications of our priority-setting results indicate a strong and urgent need to focus on well-funded research areas that have the potential to significantly reduce the burden of MNS disorders and the wide treatment gap of 98%. This could be achieved if we move beyond priority setting exercises, which are without doubt promising for surfacing vital evidence, to identify clear goals and measurable targets [10].

## Supplementary Material

Supplemental Material

## Paper context

### Evidence before this study

There are several published methods describing different approaches for setting priorities for health research. Little is known about how to strengthen standard research prioritization processes with key contextual information such as level of research activity, capacity, and funding.

### Added value of this study

This study is the first to report findings on how to use a scoping review to enhance standard research prioritization processes.

### Implications of the findings

Ghana’s context-informed research agenda provides an important framework to identify the most pressing mental health challenges.
