# Supplementary material for: Mental health and disability research priorities and capacity needs in Ghana: findings from a rapid review and research priority ranking survey
Source: Glob Health Action. 2022 Sep 29;15(1):2112404. doi: 10.1080/16549716.2022.2112404 (PMC9542869; doi:10.1080/16549716.2022.2112404)
Supplement: Supplemental Material [file ZGHA_A_2112404_SM4983.docx]

**Supplementary material**

Tables

**Supplementary Table 1: Emerging mental health research priority questions grouped in 5 thematic areas**

| **Theme** | **Description** | **No of questions identified (N=186)** |
| --- | --- | --- |
| **Thematic area 1: Epidemiological studies** | Studies on the analysis of burden, distribution, patterns and causes of mental health conditions, including social determinants of mental health | 49 |
| Thematic area 2: Intervention studies | Studies evaluating the design and delivery of mental health care; economic evaluation of mental health interventions; reducing stigma towards people with mental health disability | 32 |
| Thematic area 3: Health systems, policy, legislation studies | Studies unpacking human-rights issues and their impact on mental health; Integrating mental health services in primary healthcare; Innovative funding of mental health services | 77 |
| **Thematic area 4: public health emergencies and mental health studies** | Studies examining topics around public health emergencies such as the impact of the COVID-19 on persons with disabilities; vaccine preparedness and vaccine-related communications and engagement for persons with mental health conditions | 7 |
| **Thematic area 5: community/public engagement studies** | Studies examining the role of traditional and faith-based healers in the delivery of community mental health care and increase understanding regarding effective community participation in mental health and disability programmes. | 21 |

**Supplementary Table 2: Rationalised 55-questions**

| **Thematic area** | **Rationalised research questions** |
| --- | --- |
| **Thematic area 1: Epidemiological studies** | 1. What is the burden of mental illness and related disability in Ghana? (e.g Anxiety, Bipolar, Depression, Dementia, Epilepsy, Schizophrenia, Substance use disorders) 2. What is the impact of perinatal and postnatal mental disorders on maternal wellbeing and development of newborns? 3. What is the epidemiology of Suicide in Ghana? 4. What is the burden of mental health conditions among health workers in Ghana? 5. What is the burden of neuro-developmental problems in children in Ghana? 6. What factors contribute to relapse and resilience among people with mental illness and disability? 7. How does extreme poverty and other social determinants (including domestic abuse, childhood adversity) exacerbate mental health conditions in Ghana? 8. What is the impact of mental illness on higher educational attainment, employment and economic livelihoods in Ghana? 9. What is the prevalence, and the psychological, social and cultural determinants of mental disorders in people with physical disabilities in Ghana? 10. What are the healthcare seeking practices among people with mental illness in Ghana? 11. What is the level of mental health literacy in Ghana? 12. How is mental health stigma impacting on the economic livelihoods of persons with psychosocial disabilities? 13. What are the causes of stigma and discrimination against people with mental illness and what steps can be taken to address it? |
| **Thematic area 2: Intervention studies** | 1. What are the causes of stigma and discrimination against people with mental illness and what steps can be taken to address it? 2. Which models of mental health care are effective and cost-effective in addressing the burden of mental health conditions in Ghana? 3. What sustainable initiatives can improve community-based access to mental health services? 4. What interventions and educational programmes will aid in improving mental health literacy to reduce stigma and protecting the rights of people living with mental illness and disabilities? 5. What are the disability sensitive mental illness prevention, promotion and treatment strategies in Ghana? 6. What are the ways of improving working conditions for mental health professionals in Ghana? 7. What livelihoods and psychosocial support activities are most appropriate   and effective for people with disabilities?   1. What are the side-effects of psychotropic medicines on people with mental illness? 2. How can digital or online interventions be used more effectively to provide information and support people with mental health problems? 3. What is the efficacy of herbal medicine for the treatment of mental health conditions in Ghana? 4. What are effective strategies for preventing first onset and relapse of mental? |
| **Thematic area 3: Health systems, policy, legislation studies** | 1. What are the factors that influence good treatment outcomes for persons living with mental illness? 2. What is the extent of rights protection under current legislation and policy in Ghana for persons with disabilities, including mental disabilities? 3. How can we scale up mental health and disability services in Ghana? 4. What are the barriers and opportunities for effective societal participation (including employment) of people living with mental illness in Ghana? 5. What are the guidelines and protocols for mental health care and treatment that are sensitive to disabilities? 6. What is the impact of mental health and disability interventions (such as inclusive education of persons with disabilities) on the economy of Ghana? 7. To what extent do district/municipal assemblies include mental health issues in their short-to- long-term development plans? 8. What proportion of people with mental health and disability related health care needs access the services they require, and do not suffer financial hardship as a result? How does this differ by different groups: men/ women, rural/ urban, rich/ poor, etc? 9. How can we make mental health accessible to all Ghanaians especially those in Northern Ghana and rural areas given the inadequate health facilities in those areas? 10. What is the quality of mental health service provision for all in Ghana? 11. What are the benefits and challenges of delivering mental healthcare through community-based instead of institutional care and what impact does the removal of institutional care have on mental healthcare in Ghana? 12. How can mental healthcare be integrated in primary care in Ghana and readily accessible for all? 13. What are the best mechanisms for addressing patient safety concerns in the wards? 14. How is stigma and discriminations impacting on the social and economic lives of psychiatric doctors and nurses in Ghana? 15. How do you strengthen the capacities and collaboration among and between public sector institutions on mental health and disability? 16. Do individuals with disability enjoy equal employment opportunities in Ghana? 17. How can we ensure the rights of vulnerable groups like children with disabilities and imprisoned mental patients in Ghana? 18. To what extent has the delay in the implementation of the new Mental Health Act impacted mental health service delivery in Ghana and how can the system be strengthened to ensure persons with disabilities are included in society? 19. Are people with mental health problems and disability well engaged and involved when making policies that directly affect them? 20. What impacts will the adoption of digital technology in mental health services have on capacity, access to services, waiting times, and preferred appointment times? 21. What is the importance of community mental health officers in mental health delivery? 22. How can we improve capacity of mental health staff in Ghana, including training on disabilities and improving working conditions and what is the effect on improving service delivery? 23. What is the importance of the Disability Common Fund for people with mental and physical disabilities in Ghana? 24. What is the impact of the frequent shortage of psychotropic medication supply on mental healthcare and how can supply be improved, including through National Health Insurance? |
| **Thematic area 4: public health emergencies and mental health studies** | 1. How are persons with disabilities including people with mental health needs included in the government’s COVID-19 emergency response plan? 2. What is the impact of COVID-19 on the economic, health and social lives of people with disabilities, including mental health and substance use in Ghana? |
| **Thematic area 5: community/public engagement studies** | 1. What livelihood support programmes are available for carers and people living with mental illness and disability and how effective are these programmes in alleviating the suffering of people living with mental illness and disabilities? 2. How can people with mental health conditions and disabilities, be effectively engaged in health and social services, and what impact can their involvement have on service quality and coverage? 3. What is the role of religion, spirituality and traditional healing in mental health in Ghana? 4. What is the role of key actors including opinion leaders and government in promoting inclusive participation and protecting the rights of persons with mental health and disabilities? 5. What is the capacity of civil society organizations representing persons with disability and mental health to drive change at local and national levels? |

Figures

**Supplementary Figure 1. Number of mental health and disability studies conducted over the past 10 years (2010-2020)**
